# Supplementary material for: A new look at the architecture and dynamics of the Hydra nerve net
Source: eLife. 2024 Feb 26;12:RP87330. doi: 10.7554/eLife.87330 (PMC10942621; doi:10.7554/eLife.87330)
Supplement: Figure 10—source data 1. — The interactive 3D model can be accessed by clicking into the figure and dragging the image with the left mouse button pressed (Adobe Reader Version 7 or higher required). Scale bar: 20 µm. [file elife-87330-fig10-data1.pdf]

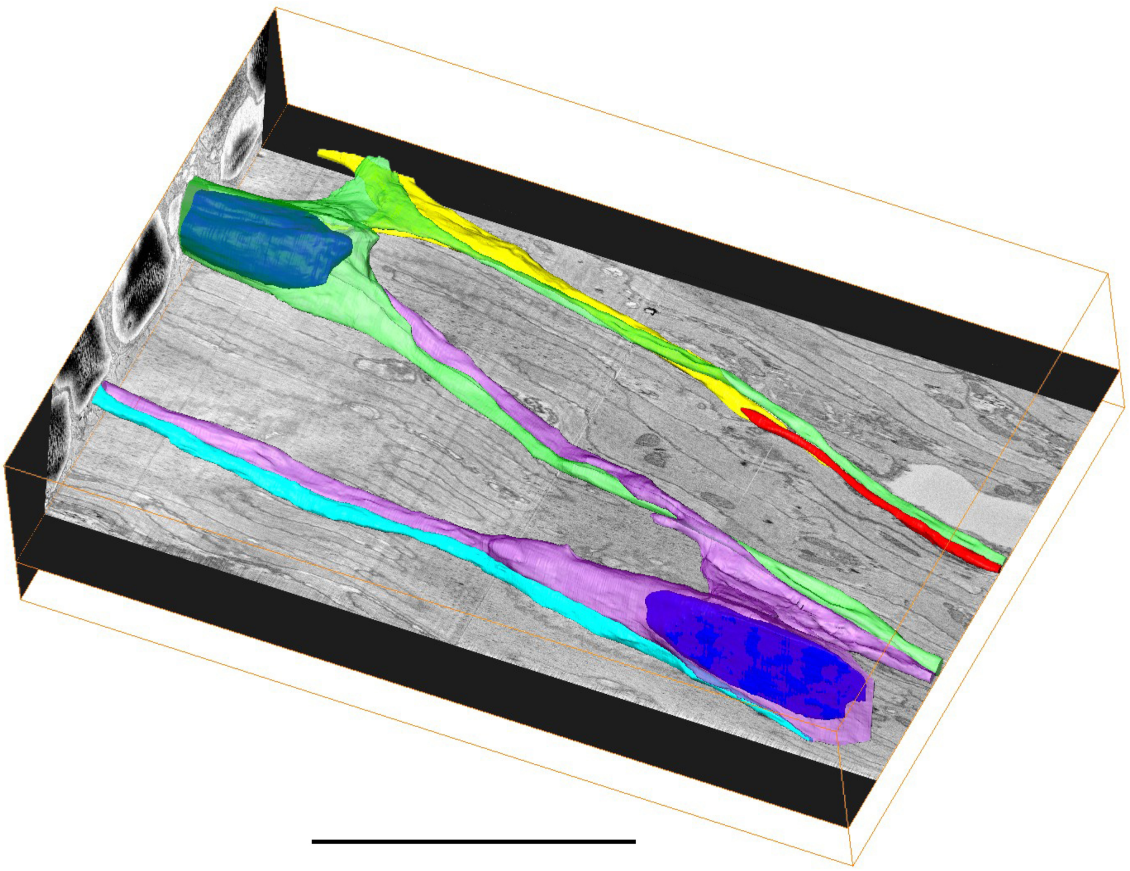

**Figure 10—source data 1**

3D reconstruction of 2000 serial sections obtained by serial block face scanning SEM in the ectoderm of the body column. Two nerve cell bodies and their neurites are colored green and purple (nuclei are highlighted in blue). Three additional neurites from nerve cells outside the imaged block are colored yellow, red and blue. The imaged block is 56 µm long. Scale bar: 20 µm.
**The interactive 3D model** can be accessed by clicking onto figure (Adobe Reader Version 7 or higher required). Rotate model by dragging with left mouse button pressed, shift model: same action + ctrl, zoom: use mouse wheel (or change default action for left mouse button). Select or deselect (or change transparency of) components in the model tree, **switch between prefab views** or change surface visualization (e.g., lightning, render mode, crop, etc.).
